# Where Are You Throwing the Ball? I Better Watch Your Body, Not Just Your Arm!

**DOI:** 10.3389/fnhum.2017.00505

**Published:** 2017-10-30

**Authors:** Antonella Maselli, Aishwar Dhawan, Benedetta Cesqui, Marta Russo, Francesco Lacquaniti, Andrea d’Avella

**Affiliations:** ^1^Laboratory of Neuromotor Physiology, Santa Lucia Foundation, Rome, Italy; ^2^Department of Biomechanics, Institute of Sukan Negara, Kuala Lumpur, Malaysia; ^3^Department of Systems Medicine and Center of Space Biomedicine, University of Rome Tor Vergata, Rome, Italy; ^4^Department of Biomedical and Dental Sciences and Morphofunctional Imaging, University of Messina, Messina, Italy

**Keywords:** biological motion perception, visual cues, predictions, inter-individual variability, overarm throwing, advanced information, dimensionality reduction, machine learning

## Abstract

The ability to intercept or avoid a moving object, whether to catch a ball, snatch one’s prey, or avoid the path of a predator, is a skill that has been acquired throughout evolution by many species in the animal kingdom. This requires processing early visual cues in order to program anticipatory motor responses tuned to the forthcoming event. Here, we explore the nature of the early kinematics cues that could inform an observer about the future direction of a ball projected with an unconstrained overarm throw. Our goal was to pinpoint the body segments that, throughout the temporal course of the throwing action, could provide key cues for accurately predicting the side of the outgoing ball. We recorded whole-body kinematics from twenty non-expert participants performing unconstrained overarm throws at four different targets placed on a vertical plane at 6 m distance. In order to characterize the spatiotemporal structure of the information embedded in the kinematics of the throwing action about the outgoing ball direction, we introduced a novel combination of dimensionality reduction and machine learning techniques. The recorded kinematics clearly shows that throwing styles differed considerably across individuals, with corresponding inter-individual differences in the spatio-temporal structure of the thrower predictability. We found that for most participants it is possible to predict the region where the ball hit the target plane, with an accuracy above 80%, as early as 400–500 ms before ball release. Interestingly, the body parts that provided the most informative cues about the action outcome varied with the throwing style and during the time course of the throwing action. Not surprisingly, at the very end of the action, the throwing arm is the most informative body segment. However, cues allowing for predictions to be made earlier than 200 ms before release are typically associated to other body parts, such as the lower limbs and the contralateral arm. These findings are discussed in the context of the sport-science literature on throwing and catching interactive tasks, as well as from the wider perspective of the role of sensorimotor coupling in interpersonal social interactions.

## Introduction

A smooth and successful interaction with the dynamic environment in which we live requires predictive abilities. This is true when interacting with inanimate moving objects (Lee, 1980; Zago et al., 2008), when moving through space (Regan and Gray, 2000; Hayhoe et al., 2012), as well as when interacting with other humans (Baldwin and Baird, 2001; Sebanz and Knoblich, 2009; Ambrosini et al., 2015) or with robotic agents (Gielniak and Thomaz, 2011; Takayama et al., 2011; Sciutti et al., 2015).

Interceptive sports that involve striking, hitting or catching fast moving balls serve as excellent examples of actions in which predictive strategies are a pre-requisite for successful performance (van der Kamp et al., 2008; Müller and Abernethy, 2012). Within this context, information available for prediction can be classified into two different categories: (i) information associated with the ball trajectory after its separation from the end effector (Peper et al., 1994; Regan, 2012) and (ii) information embedded in the kinematics of the throwing action, also known as advanced information (Farrow et al., 2005; Müller et al., 2006).

Precise predictions can be made based on the ball flight after ball release. These are used for compensating sensorimotor delays in interceptive tasks (Davidson and Wolpert, 2005; Zago et al., 2008) as well as for the fine-tuning of grasping control in catching (Cesqui et al., 2016). However, depending on temporal and/or visibility constraints, information from the ball flight alone does not always allow formulating predictions with sufficient anticipation so as to successfully intercept the ball trajectory. Therefore, any other information available to the catcher prior to ball release that contributes to the estimate of ball path and kinematics, could be used to enhance interceptive performances.

Sport science literature has shown that expert players are able to pick up advanced information from an observed throwing action (Abernethy, 1990b; Abernethy et al., 2008; Aglioti et al., 2008) and to exploit such information for optimizing their interception/catching performances (Ranganathan and Carlton, 2007; Renshaw et al., 2007; Mann et al., 2010). More specifically, it has been shown that advanced information from the throwing kinematics triggers anticipatory motor responses, such as directing the end-effector or displacing the whole body toward the region of space in which the ball is predicted to flight through (Abernethy, 1990b; Mann et al., 2010). Nevertheless, the nature of the informative kinematic cues allowing for such predictions is not well known (Müller et al., 2006). Furthermore, because of the strong focus on the role of expertise in sport science studies, the predictive skills of non-expert participants have not been sufficiently tested yet, if not for the case of predictions based on the initial ball trajectory (Cesqui et al., 2015).

Beside the context of elite-sports performances, throwing and catching are fundamental skills in the human motor repertoire (Haywood and Getchell, 2014), and it is reasonable to assume that predictive mechanisms are involved in the execution of interceptive tasks independently on the temporal constraints intrinsic of fast-ball sports. In fact, recent research has shown that predictive mechanisms elicited by the observed body kinematics seamlessly shape interpersonal interaction in a number of standard social interactive activities (Sartori et al., 2011; Pezzulo et al., 2013; Ansuini et al., 2014). Reliable predictions about the intention and outcome of an observed action can indeed be made because action execution is itself shaped by its final goal and/or by the physical properties of its target. For example, the kinematics of the arm and the fingers in a reach-to-grasp action is modulated by the size and weight of the object to be grasped (Berthier et al., 1996; Eastough and Edwards, 2007), and changes if one reaches for a bottle to pour its content or to pass it to someone else (Ansuini et al., 2008). The key point is that, because the action is shaped by its final goal, information about the goal is available to the observer before action execution is completed. Importantly, this applies to a wide spectrum of ecological motor behaviors, from simple actions like reach-to-grasp an object, to more complex ones like throwing a stone to hit a prey or playing an interactive ball game.

In a recent proposal, Ansuini and colleagues suggested to approach the problem of intention-from-movement understanding taking “action execution” as a starting point (Ansuini et al., 2015a). First, the extent to which intentions and goals shape the spatiotemporal features of action execution must be characterized. Subsequently, this knowledge can be used to assess whether and how an observer is able to read the available information (cues) and to exploit in the planning of adequate counteractions (Ansuini et al., 2015a). We have adopted this principled approach to determine which visual cues from an overarm throwing action are the most informative about the outgoing ball direction and could therefore guide the interceptive behavior of a catcher.

In this study, we take on the issue of characterizing the information embedded in the kinematics of unconstrained overarm throwing actions in relation to the outgoing ball direction. We asked twenty non-expert participants to throw a ball aiming at different targets on a vertical board placed at 6 m distance. By analyzing full body kinematics off-line, we looked for the cues about ball direction that are available from the thrower’s kinematics prior to ball release. Our long-term goal is to verify whether these visual cues are used by human catchers in interception tasks.

We designed our analysis to perform a coarse spatial decoding of the region of space where the ball arrived (Right vs. Left). In doing so, we adopted a classification approach, using Linear Discriminant Analysis (LDA), to assess how well different subsets of kinematic predictors from the throwing action are able to discriminate whether the ball will fly toward the right or the left side. This choice is in line with the evidence that, during an interceptive task, advanced information from a throwing action is typically coupled to gross-movement responses functional for the catcher to reach the region of space in which the ball is predicted to arrive, so as to optimizing successful performances (Abernethy, 1990b; Mann et al., 2010).

In order to address the issue of *how early* useful information is available for a potential observer to make reliable predictions about the ball’s flight, and to determine how such early information is spatially structured in terms of specific visual cues from the whole-body kinematics, we applied LDA classification using different sets of predictors. These include the kinematics of individual markers placed on single joints across the whole participant’s body, and of sets of multiple joint-markers, across different temporal windows throughout the unfolding of the throwing action. In doing so, as an intermediate step, we adopted a spatiotemporal principal component analysis (stPCA) to obtain a compact description of the considered kinematics.

The rationale for the choice of the described approach is rooted in the expectation that the obtained results would guide us in identifying, unbiasedly, the spatiotemporal structure of early information embedded in throwing actions, and in establishing how this structure depends on individual throwing styles. On the long-term this outcome would be useful for exploring to which extent humans are able to access and use the available visual cues, as well as for designing artificial agents that optimize their predictive abilities by exploiting previous knowledge about the spatiotemporal structure of the information available.

## Materials and Methods

### Participants

Twenty right-handed participants (10 females, 10 males; age: 28.2 ± 6.8 years), with normal or corrected-to-normal vision and no history of neurological conditions, participated in the experiment. Handedness was tested with the standard Edinburgh Handedness Questionnaire (Oldfield, 1971). Upon analysis of the handedness questionnaire, eighteen participants were classified as right handed [Lateral Index (LI): 84.8 ± 6.8]. The remaining two participants were classified as ambidextrous (Laterality Index: LI = 30 and LI = 26. Data from all twenty participants were included in the analysis. All participants read and signed an informed consent prior to their participation and received a monetary compensation proportional to the full duration of the experiment.

This study was carried out in accordance with Italian laws and European Union regulations on experiments involving human participants. All subjects gave written informed consent in accordance with the Declaration of Helsinki. The protocol was approved by the Ethical Review Board of the Santa Lucia Foundation.

### Apparatus

A sixteen camera opto-electronic system (OptiTrack, NaturalPoint, Inc., Corvallis, OR, United States) operating at 120 Hz was utilized to capture full body positional information of the participants throwing actions and the corresponding ball trajectories. Infrared cameras were strategically located to allow for a large and gap-free calibration volume of 10 × 6 × 3 m^3^. All positional data were reconstructed by a dedicated software (Motive, Optitrack, Natural Point, Inc., Corvallis, OR, United States). To minimize marker reconstruction artifacts, participants were required to wear a body fitted velcro suit and a beanie cap, upon which retroreflective markers (diameter 14 mm) were attached. Each participant was equipped with a standard biomechanical marker set consisting of 57 retroreflective markers. Eight of these markers, located on medial anatomical joints, were used only for calibration and were removed post calibration procedure (Supplementary Figure 1). The marker set allowed for a real-time skeleton animation of the moving participant and for saving data with automatically labeled marker trajectories. A customized foam ball (40 g, 90 mm diameter) was used as a throwing object. The ball was embedded with five asymmetrically located retroreflective markers and subsequently a rigid body was created to track the ball trajectory.

Participants had to perform a series of overarm throws, starting from a fixed initial position. They were instructed to hit one of four circular targets arranged on a vertical target board placed at 6 m from the initial position (marked with a sign on the floor) and to start from a fixed posture (standing with the arm along the body; as in Supplementary Figure 1). The four targets were custom made and consisted in white circles of 40 cm diameter, arranged on a rectangular layout on the target board. The distances between the centers were 70 cm vertically and 80 cm horizontally. Moreover, the targets midpoints in the horizontal direction were shifted with respect to the projected participant’s initial position: the left and right targets were centered respectively at 60 cm to the left and 20 cm to the right of the projected initial position of the thrower’s midline.

**FIGURE 1:**
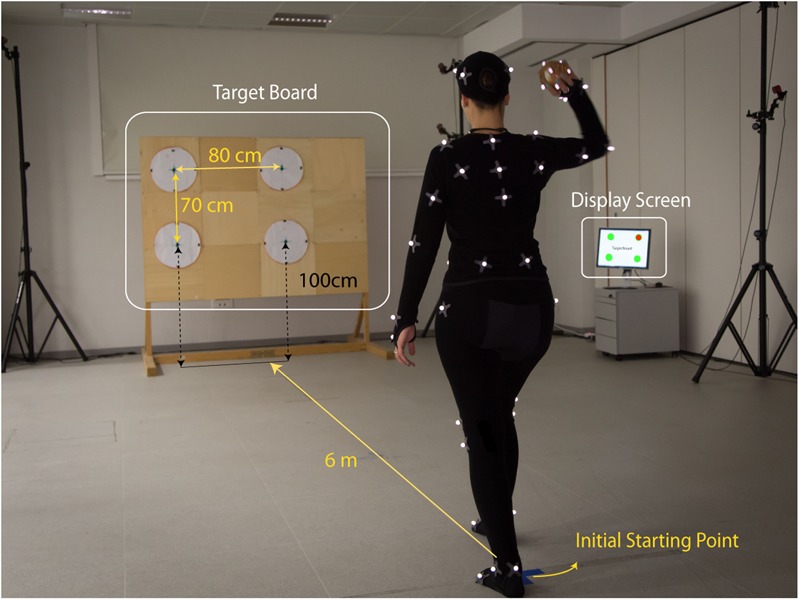
Experimental setup. A motion capture OptiTrack system with 16 infrared cameras was used to record the kinematics of 20 participants performing overarm throws. The participants, instrumented with a set of retroreflective markers, performed free overarm throws aiming at one of the four targets arranged on a wooden board (*Targets Board*) placed at 6 m from the initial starting position of the thrower. At the beginning of each trial, the actual target to aim for was indicated on a *Display Screen*. For each trial, the motion capture from the thrower kinematics and the ball flight was recorded for a duration of 6 s. The picture shows author MR performing a throw in the experimental setup. Author MR gave her consent for image publication.

The beginning of each trial was signaled with a computer-generated pure tone sound and by displaying the selected target on a computer monitor (see **Figure 1** – Display Screen). A second pure tone sound marked the end of the trial after 6 s. In addition, a USB video camera was located outside the capture volume (see **Figure 1** – video camera) and recorded the target location, the participant throwing action and the ball trajectory, including its landing location on the target-board, for the full duration of the trial. The handling and communication of all of the above information was managed through a custom Matlab script that integrated Optitrack Natnet SDK^1^. The Natnet.NET client/server architecture allowed seamless communication between Motive and Matlab (v2014b), allowing for control via Matlab of Motive commands (e.g., start, stop, capture a new trial, import, etc.). The connection to the display computer was established using a UDP connection.

### Protocol

Participants were instructed to perform unconstrained overarm throws to hit one of the four targets on the targets-board, as indicated on a display at the beginning of each trial.

Participants were first instrumented with the 57 retroreflective markers for motion capture, a procedure that took about 10 min on average to be completed. After marker instrumentation, participants performed a standard warm-up session which required them to (i) perform multiple overarm shadow throws (without the ball), (ii) perform overarm throws with the ball aiming to hit the rectangular board and, lastly, (iii) perform a minimum of 12 throws, 3 toward each circular target. Participants were then allowed to train further, until they felt at easy with the task. The training phase lasted about 5–7 min. The experimental recording session was initiated right next and had an average duration of 40 min.

The experimental session consisted in a series of 120 throws, 30 for each of the four targets, presented in a pseudorandom order. No instruction was given regarding how to perform the throwing movement, nor feedback about performances during the experiment. To start from a fixed initial position, facing the targets-board and standing straight, with the arm along the body and the hands slightly separated from the thighs. Participants were therefore free to perform a step forward while throwing (with either of the two legs) or to throw standing at place. The session was divided into three blocks of 40 trials, although participants could ask at any moment to take a break if needed.

### Data Processing and Analysis

The positional data of a subset of eighteen markers selected from the Motive biomechanical markerset and the positional data extracted from the ball trajectory after ball release were analyzed in this study. The subset of eighteen markers included left and right metatarsal (foot), lateral malleolus (ankle), femur epicondyle (knee), iliac crest (pelvis), acromion (shoulder), humerus epicondyle (elbow), ulna syloid process (wrist), second proximal phalanx (hand), seventh cervical vertebra (cv7) and anterior head (Supplementary Figure 1). The head’s positional data were obtained as the mean of the right and left anterior head markers.

Differently from the joint-markers, the ball was tracked as a rigid body composed of five markers arranged asymmetrically on the ball surface so to facilitate its grasping. Consequently, the centroid of the rigid body did not perfectly coincide with the center of the ball. The positional data of the ball center were then estimated by fitting, at each recording frame, the available ball markers positional data onto a sphere of known radius (45 mm).

Altogether, data from 2400 throwing trials were recorded, 120 for each of the 20 participants.

### Data Processing

For each trial the positional data from the eighteen joint-markers selected for the analysis of the throwing motion, as well as the ball center positional data, were first interpolated using a cubic spline method (to fill recording gaps), and subsequently filtered using a zero-lag Butterworth filter of order 5 and low pass frequency 15 Hz (Matlab *filtfilt* function). Positional data were then differentiated (Matlab *diff* function) to obtain velocities and accelerations.

Due to the challenging setup, which required to track the thrower’s whole-body movements together with the ball flight over a large tracking volume, several trials were excluded due to poor tracking. Exclusion criteria were of three categories. First, if one or more of the joint-markers had been assigned with impossible values or had no recordings assigned, which occasionally happened when the skeleton reconstruction was not successful. Second, if the right-hand tracking was lost at ball release, which occasionally happened as the ball markers got confused with the markers on the hands, so that the skeleton reconstruction of the arm temporarily failed and the ball rigid body was lost. Third, if the ball recordings had more than 25% missing frames in the motion capture between ball-release and ball-board impact (see below). Typically, recording data along the ball flight trajectory were missing when the ball flew close to the ceiling, at the boundary of the capture volume, or in the proximity of the target-board, as the latter introduces occlusions that limit the cameras field of view in its vicinity. Additionally, a few trials were excluded when the participants failed to follow the instruction and performed a step before starting the genuine throwing action (e.g., repositioning the starting position).

All the data processing, including the automatized exclusion of bad-trials and the identification of key-events (see below) were performed with custom-made software coded in Matlab. Overall, 13.8% of the trials were excluded (331 over 2400). On average, 103.5 ± 14.5 good trials per participant were used for the analysis. Individual data is listed in **Table 1**.

**Table 1:** Average throwing performances and analysis parameters for individual participants.

Participant ID	Success Rate [%]	Throw duration mean ± SD[s]	Flight time mean ± SD [ms]	Release speed mean ± SD [m/s]	Trials used for PCA	Trials used for LDA	Classification error from ball [%]
1	25.2	1.61 ± 0.29	593 ± 61	10.56 ± 0.72	105	81	2
2	31.4	1.04 ± 0.18	661 ± 41	10.38 ± 0.48	111	88	0
3	12.5	1.04 ± 0.23	694 ± 61	9.72 ± 0.55	103	82	1.2
4	65.5	0.81 ± 0.07	557 ± 37	11.39 ± 0.52	117	108	0
5	33.1	1.12 ± 0.10	590 ± 37	10.16 ± 0.66	117	106	0
6	18.5	1.41 ± 0.12	570 ± 59	10.02 ± 0.50	111	89	0
7	31.7	1.00 ± 0.32	439 ± 37	12.90 ± 0.91	79	64	0
8	31.1	0.92 ± 0.13	567 ± 43	11.13 ± 0.66	90	76	0
9	24.1	1.13 ± 0.22	762 ± 11	8.77 ± 0.92	89	71	4.2
10	19.3	1.02 ± 0.21	735 ± 64	8.88 ± 0.37	116	92	1.1
11	19.5	1.73 ± 0.32	421 ± 44	13.64 ± 1.18	114	94	3.2
12	20.8	1.27 ± 0.15	567 ± 56	10.56 ± 0.47	113	102	0
13	42.0	1.84 ± 0.26	642 ± 67	10.10 ± 0.65	107	93	0
14	25.0	0.95 ± 0.30	443 ± 49	13.85 ± 1.23	96	73	0
15	26.9	1.25 ± 0.12	647 ± 64	10.46 ± 0.54	110	88	1.1
16	25.0	1.05 ± 0.15	705 ± 46	9.18 ± 0.35	60	52	0
17	27.7	1.23 ± 0.27	643 ± 93	9.92 ± 0.75	107	87	1.1
18	60.8	1.24 ± 0.20	498 ± 51	11.97 ± 0.71	102	99	0
19	17.8	1.70 ± 0.36	731 ± 62	9.00 ± 0.37	115	92	2.2
20	21.0	1.27 ± 0.36	623 ± 81	9.98 ± 0.57	107	86	2.3
All (mean)	29.0	1.23 ± 0.36	603 ± 11	10.63 ± 1.59	103 ± 14	86 ± 14	0.9 ± 1.3


Grand-averages are listed at the table bottom.

#### Identification of Key-Events

For each trial, we identified three key events: throwing action onset, ball release and ball-impact on the target-board.

The *throwing action onset* was defined as the onset of the throwing hand initial rising movement. The procedure consisted of the following steps. First, the time at which the hand first rose 5 cm above the hip was identified based on the hand-hip distance profile. Next, the onset detection was based on the speed profile of the throwing hand in a 500 ms interval centered on this event. This was done to remove movements that occasionally participants performed before the actual throwing action, e.g., swinging arms. The throwing onset was then defined as the point at which the speed profile intercepts its tangent taken at the point where the speed reaches 20% of the maximum speed in this interval (see inset in Supplementary Figure 2).

The exact time of *ball release* is the time at which the ball reaches its maximum speed. In fact, once the ball is released, it is no longer accelerated by the throwing hand and its speed starts decreasing. However, prior to release, the ball is poorly tracked as its markers are partially occluded by the holding hand, and it is not possible to reliably extract its speed profile. Instead, we considered the hand speed, which is coupled with that of the ball up to the time of ball release and start decreasing at the time of ball release. The time of ball release was then estimated as the time at which the right (throwing) hand speed profile reached its maximum value in the 500 ms interval centered on the time at which the hand-ball distances was above a given threshold. Such threshold was set according to the hand-ball distance calculated at the beginning of the trial, i.e., when the ball was held in the hand (see Supplementary Figure 2).

The identification of the *ball-impact* event was based on an extrapolation procedure. The ball trajectory was first fitted with a second order polynomial function in the time interval [*150 ms, 450 ms*] starting from ball release, and then extrapolated for the subsequent 300 ms. Extrapolation of the ball trajectory was required because the targets-board occluded the field of view of some of the cameras, resulting in a poor tracking of the last part of the ball trajectory in a large fraction of the trials.

### Analysis

#### Linear Discriminant Analysis for Throwing Outcome Classification

We conducted Linear Discriminant Analysis (LDA) on different sets of kinematic variables extracted from positional data with the aim to quantify the predictability of a throw’s outcome. Among a variety of possible classifiers, we adopted LDA for several reasons. First, in this study we were mainly interested in the spatiotemporal structure of the classification accuracy, rather than in the precision of the corresponding absolute values. Non-linear classifiers, with the drawback of increasing the level of complexity, could possibly result in an overall higher classification accuracy (Amancio et al., 2014; but see Kim, 2008), but it is unlikely that the overall spatiotemporal structures would be significantly affected. Second, with respect to other linear classifiers such as linear regression or linear support vector machine algorithms, LDA can be easily extended to multi-classes (higher than two-classes) problems, which can be useful in future extension of the present work, e.g., for multi-targets classification.

The kinematic variables included are the joint-markers positions and velocities. Although it has been shown that accelerations play a critical role in the perception of biological motion (Troje and Westhoff, 2006; Chang and Troje, 2009), the visual system is known to be poor at directly discriminating accelerations from an observed moving object (Calderone and Kaiser, 1989; Werkhoven et al., 1992; Brouwer et al., 2002). As a trade-off, we included information on accelerations only implicitly, namely in the form of the veridical temporal structure of the joint-marker’s position and velocity. This choice was furthermore functional to limiting the dimensionality of the predictor space, which would otherwise increase importantly.

LDA is a standard classification technique used to allocate a new observation to one of *N_G_* ≥ 2 groups (usually referred to as classes) based on its measured parameters (features). LDA consists in finding discriminant functions that divide the features space into *N_G_* regions, so that each region spans the range of features values that most likely characterize a given class. This is done on the base of a set of previous observations for which the group assignment is known (training dataset), by maximizing the ratio of the between-groups to the within-group variabilities (Mardia et al., 1995).

**FIGURE 2:**
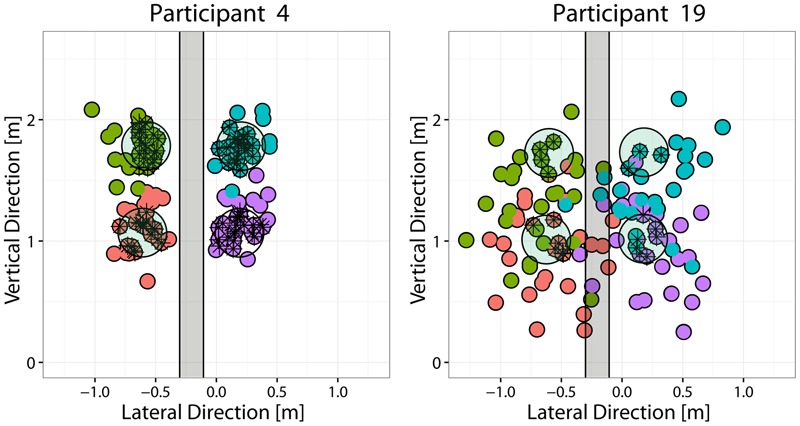
Throwing performance of the best and worst performing participants. Each panel depicts the targets-board with the four targets (light-green circles), together with the distribution of the points where the ball hit the targets-board (small colored circles) for the best (P4, left) and the worst (P19, right) performing participants. Different colors are used to indicate the target that the participant was instructed to hit. For the classification analysis, the throws were instead grouped into two different classes (Right vs. Left) according to the throw’s impact point coordinates. The area shaded in gray (22 cm in width) represents the “gray” region separating the two classes: throws that reached the targets-board in that region were discarded from the LDA analysis.

Because the focus of this study was to examine the nature of advanced visual cues that allow making reasonable predictions about the outgoing ball direction prior to ball release, classes were defined according to the region in which the outgoing ball landed on the targets-board. For the analysis presented in this paper, throws were grouped according to the *Side* (Right vs. Left) of the ball’s landing position with respect to a separation region. The latter was defined as a vertical band centered at the lateral (*x*) coordinate corresponding to the mean *x* across all trials (therefore participant dependent) and having a width equal to 25% of the lateral separation between the targets, i.e, 22.5 cm (see **Figure 2**). This choice allowed including in the analysis most of the performed throws, even for the poorly performing participants and, at the same time, to have an even repartition of the trials in the different classes.

As the inter-individual variability observed in throwing styles was way larger than the intra-individual variability associated with throws to different targets, the analysis was conducted independently for each participant. For each participants, we considered the kinematics across a temporal window of fixed duration, equal to the individual’s average throwing duration (from throwing onset to ball release, see **Table 1**), extending up to the time of ball release. The trials of equal duration were then aligned at the end of the time window, i.e., at ball release, and resampled on 100 time points.

We adopted an exploratory approach. LDA was indeed performed on different sets of predictors. We considered kinematic variables (positions and velocities) from single joint-markers, and from subsets of joint-markers representing specific body parts, over different phases and durations of the throwing motion. When defining subsets of joint-markers we considered six different body districts: trunk (including right and left shoulder and hip), head (including anterior head and cv7 markers), right and left arms (including respectively right and left elbow, wrist and hand), and right and left legs (including respectively right and left keen, ankle and foot). For both single joint-markers and body-parts, we performed the analysis on ten time intervals (deciles), and for each of them we considered two different subsets of data: Position and velocity were either monitored across the duration of the corresponding decile (*time-decile* case: non-overlapping time intervals each including 10 time points), or during the whole duration of the action from action onset to the end of the corresponding decile (*time-through* case: overlapping time intervals of increasing duration, including from 10 to 100 time points). Our main analysis has been performed on the *single joint-makers*/*time-decile* datasets. The other datasets have been considered to address the question of whether integrating information across time and space allows making better predictions, or is instead detrimental because it cumulates non-informative variance that weaken classification performances.

When considering the kinematics of subsets of joint-markers over extended time periods, the dimensionality of the predictors space could increase dramatically (Supplementary Table 1), reaching a maximum of 2400 features for the case of the “trunk” body part in the last decile of the *time-through* case (6 position-velocity coordinates × 4 joint-markers × 100 time samples). It was therefore necessary to adopt a dimensionality reduction technique to obtain a compact representation of the considered kinematics, in order to run LDA with the limited number of trials available. A similar two-step PCA/LDA approach has been previously adopted in the literature, e.g., for facial expression recognition (Calder et al., 2001), and proved to be robust for efficient classification/recognition problems (Yang and Yang, 2003).

In the following, LDA results are reported in terms of the misclassification errors (MEs) resulting from a leave-one-out cross validation procedure. The latter consists in performing the classification assignment of a single observation (the one left out) based on the training set defined by the rest of the observations, repeating the same procedure for all observation, and computing the percentage of misclassified observations.

#### Dimensionality Reduction via Spatiotemporal Principal Component Analysis

In order to obtain a compact representation of the individual throwing kinematics, we adopted spatiotemporal Principal Component Analysis (stPCA) (Russo et al., 2014). To perform stPCA, the feature space should be defined by all the temporal samples of the kinematic variables considered. For example, in the case of considering position and velocity of a single joint-marker during one time-decile, each trial will be represented as a column vector containing all the 10 temporal samples for each of the six position and velocity coordinates, so 60 features. In general, when considering the kinematics in terms of position and velocity of an arbitrary set of *N_J_* joint-markers across an arbitrary temporal interval of *N_T_* time samples, the features vector for a single observation (i.e., a throw trial) will be: q = [S_j_(t_i_),v_j_(t_i_)] ∀ j ∈ [1,N_J_] ∧ t_i_ ∈ [1,N_T_].

The features space is then of dimension N_F_ = 3 × 2 × N_j_ × N_T_. The whole set of N_Trials_ observations can be then represented by stacking the features vectors representing single trials into a single N_F_ × N_Trial_ matrix, X = [x_1_ x_2_...x_NTrials_]. Standard principal component analysis (PCA) is then applied to this matrix *X*. The PCA returns a new set of N_F_ features vectors, the principal components, defined as the linear combinations of the original features that maximize the variance observed in the datasets, ordered as function of decreasing variance accounted for (VAF). In this way it is possible to describe each observation as a linear combination of spatiotemporal principal components (stPCs) and, by neglecting a small part of the variance in the dataset, to use a limited number of stPCs for obtaining a *parsimonious summarization* of the observed datasets: x_k_ ≃ $\sum_{\mathrm{l=1}}^{N}$ c_l,k_ p_l_, with N < N_F_ and k ∈ [1,N_Trials_].

The advantage of adopting a spatiotemporal description of the kinematics, instead of a classical time-dependent spatial description (Troje, 2002; Daffertshofer et al., 2004; Huys et al., 2008), is that the temporal dependence is embedded in the principal component vectors, which indeed represents complex spatiotemporal positional and velocity trajectories for all the joint-markers considered. As a result, the description of the observed kinematics in terms of these spatiotemporal PCs (hereafter stPCs) does not involve the temporal dependence and can be extremely compact. For example, the *time-through* description of kinematics of the *trunk* body-part in the last decile of the throwing action, represented by 2400 scalars in the original feature space, can be described with less than 31 parameters in the stPCA space while accounting for 98% of the total variance (average across participants).

## Results

### Average Performances

Average performances in terms of success rate, i.e., the percentage throws in which the ball landed within the 20 cm disk of the instructed target, varied across participants from a minimum of 20% to a maximum of 66%. Although the success rate could vary with the targets location, there was no common trend across participants. **Figure 2** shows the distribution of ball landing positions on the targets-board, for the best and worst participants in terms of performance. In the cases of poorly performing participants, the distribution of the ball-board impact points was broad (e.g., P19) and there was no segregation of the trials intended to hit different targets (as in the case of good participants, e.g., P4), but instead a substantial degree of overlap among them.

Data revealed a great variability in the duration of the throwing action, in the ball release speed and in the corresponding duration of the ball flight, particularly across participants. This variability reflects the fact that participants have been not assigned with specific constraints for the throwing task execution (see Section Protocol). **Table 1** reports the summary statistics for these parameters at both the individual and the population levels.

### Throwing Styles

**FIGURE 3:**
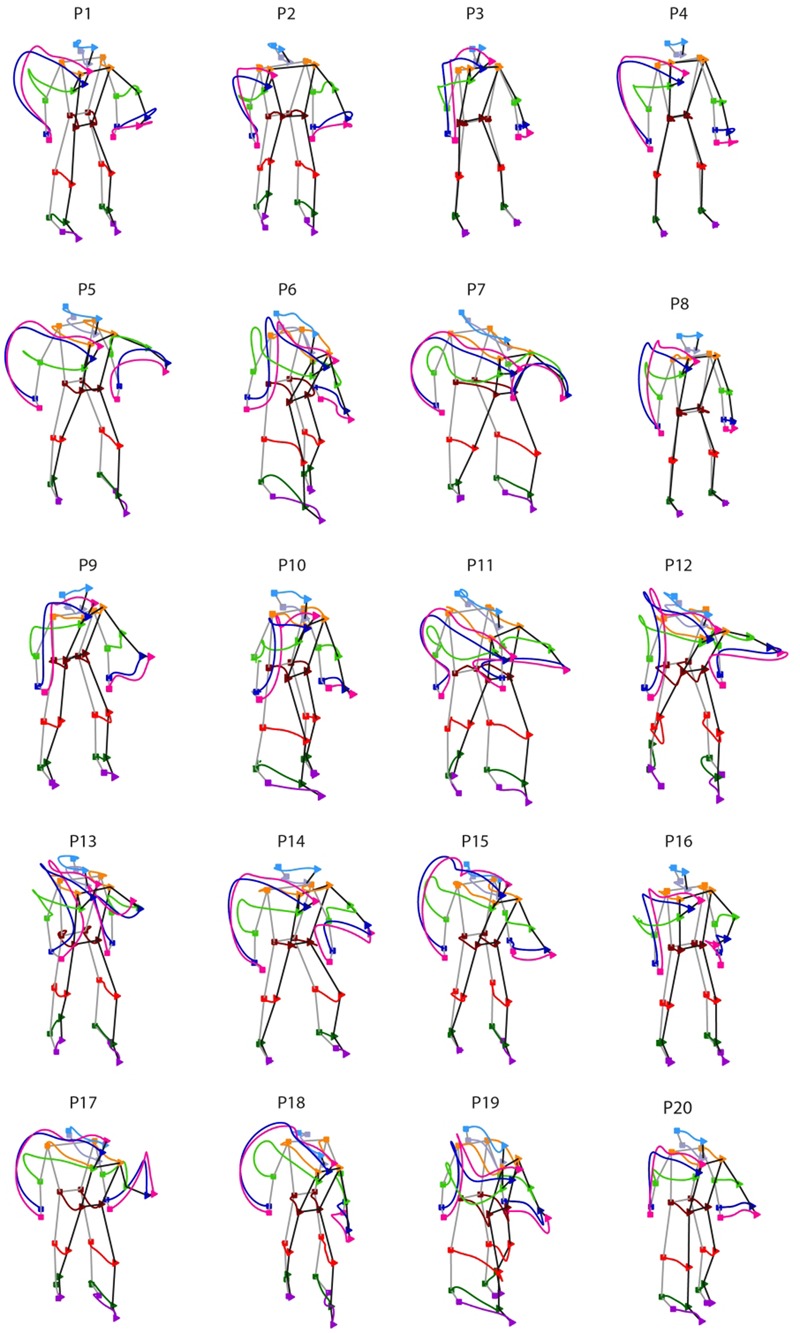
Inter-individual variability in throwing styles. Individual throwing styles are shown for the 20 participants recruited in the study. For each thrower, the displayed curves represent the averaged joint-markers trajectories across all trials performed. Square and triangle symbols indicate respectively the beginning of the throwing action and the time of ball release. Remarkable inter-individual differences are observed in the participants sample.

We next inspected the individual throwing style, namely the spatial features of the whole-body kinematics that characterizes an unconstrained overarm throwing action. **Figure 3** shows the individual styles from the 20 participants recruited in the study. For each participant, the figure depicts the trajectories of the joint-markers included in the analysis averaged across all trials performed, independently on the target aimed for. The inter-individual differences in throwing styles are markedly evident.

Despite the large inter-individual variability in throwing styles, for all participants the throwing action can be clearly segmented into two phases: one in which the throwing hand is elevated at about the shoulder level (*elevation*) and the other in which the hand is projected forward for executing the throw (*forward-projection*). During *elevation*, the throwing hand trajectory may have two main modes of execution. Some participants rise the throwing hand by moving the arm in front of the trunk (e.g., P3, P9, P16, P19) and in some cases slightly toward the body midline (e.g., P6, P10, P12, P13). Otherwise, the throwing hand is risen while moving backward and opening laterally towards the right side (e.g., P1, P4, P5, P7, P14, P15, P17, P18). Other important inter-individual differences can be appreciated in the stepping. Some participants adopt an “on-place” throwing strategy keeping the feet at place (e.g., P3, P4, P8), while others undertake one prominent step, either with the left (e.g., P7, P17, P18) or with the right (P6, P10, P19) leg. Variability is there also in the degree of the trunk’s forward projection and in whether and how the left arm is involved in counterbalancing the throwing arm motion.

**FIGURE 4:**
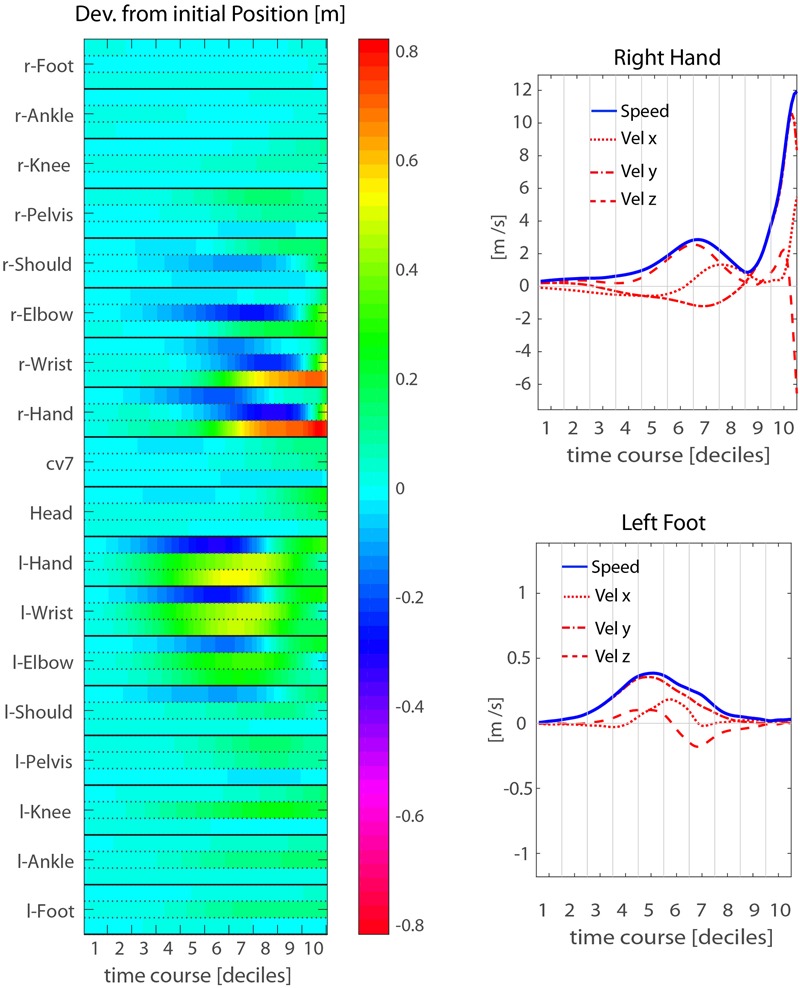
Mean throwing action kinematics of representative participant P14. (Left) The temporal evolution of the deviations from the initial position is shown for all joint-makers included in the analysis. The three rows associated to each joint-marker represent the values for the three spatial coordinates, [x,y,z] from top to bottom respectively. (Right) Examples of velocity and speed profiles from single joint-markers: right hand (upper) and left foot (lower). The participant’s throwing style is characterized by an *elevation* phase in which the throwing arm is risen while moving the hand backwards and to the right side, and by a concomitant left step forwards. While the elevation phase extends in time throughout the first eight deciles of the whole action duration, the *forward-projection* phase occurs late, across the 9th and 10th deciles.

A more detailed, though less intuitive, description of the throwing action kinematics is provided by the heat-map representations of **Figure 4**, showing the kinematics of a representative participant (P14). The map in the left panel represents the deviation from the corresponding initial values of the three positional coordinates (x, y, z) of each joint-marker through time. For this particular participant, the action starts with moving the right arm backward, while the hand and wrist start rising. This is compensated by left arm moving forward (positive displacements along the *y*-axis), and soon after is followed by a left step forward resulting in a trunk rotation, with right side moving backward and the left forward. All these kinematic features unfold during the *elevation* phase. The *forward-projection* phase starts only at the very end of throwing action, i.e., during the 9th decile, about 200 ms before ball release.

**FIGURE 5:**
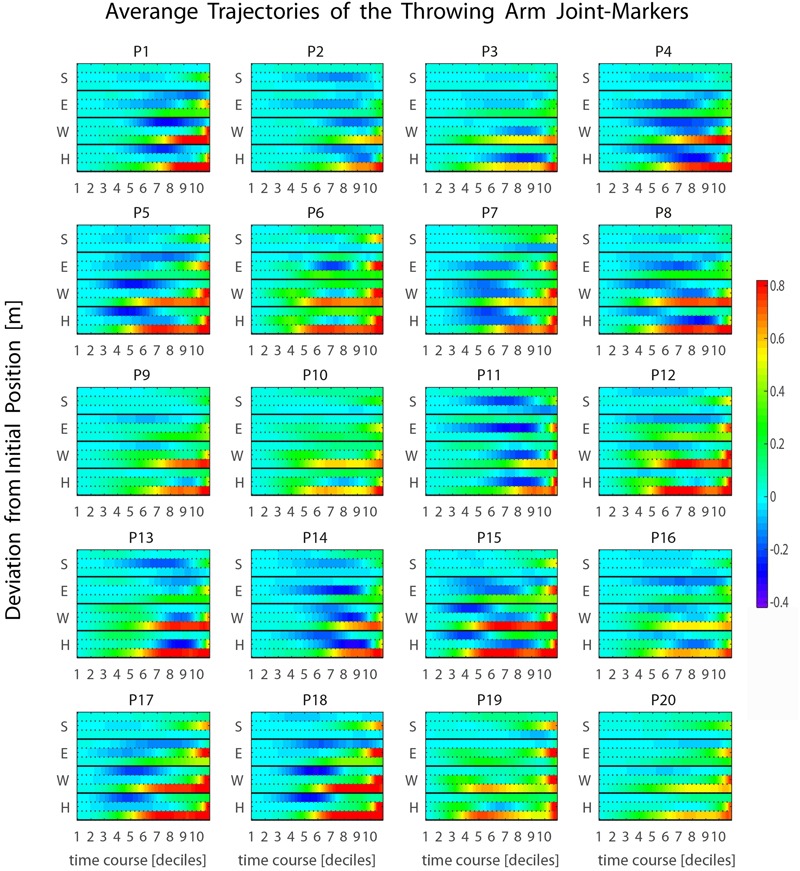
Average kinematics of the four throwing arm marker-joints. (S, shoulder; E, elbow; W, wrist; H, hand) for the 20 participants recruited in the study. The heat maps represent the temporal evolution of the deviation from the initial position through time. The three rows associated with each joint-marker represent the values for the three spatial coordinates, [x,y,z] from top to bottom respectively. For all participants the forward-projection phase occurs typically across the 9th and 10th deciles, i.e., about 200–100 ms before ball release.

Such a late transition from the *elevation* to the *forward-projection* phase is observed for all participants in our sample (see **Figure 5** and Supplementary Figure 3), and typically occurs during the 9th or the 10th decile, about 200–100 ms before ball release. This has important implications. In fact, the throwing arm kinematics during the *forward-projection* phase is expected to be highly informative about the outgoing ball trajectory. However, such relevant information becomes available at a late stage. The question of whether other body parts may provide earlier information about the outgoing ball trajectory, even if with less precision, is therefore of particular interest. The analysis presented in the following sections addresses the issue.

### Ball Flight Predictability Based on Throwing Kinematics

In order to explore how early and which kinematic cues allows to make accurate predictions about the region in which the ball is going to land, we applied LDA to the kinematics of individual joint-markers, or subsets of joint-markers, taken across different time intervals.

As a first exploratory step, we considered predictions associated with the kinematics of individual joint-markers at 10 non-overlapping time intervals of equal duration spanning the whole throwing action (*joint-marker/time-decile*). Next, we assessed the impact of integrating advanced information across time (*joint-marker/time-through*) and/or across space (*body-parts/time-decile* or *body-parts/time-through*), by comparing prediction accuracies between the different predictor spaces considered, i.e., between different combinations of spatial and temporal information included in the LDA analysis.

Before moving on presenting the main results that quantify prediction accuracies, we shall discuss the implications that our choice of defining discrete classes (Right vs. Left), on the basis of continuous variables (coordinates of the ball landing position on the target board), has on the intrinsic accuracy of the LDA results.

#### Intrinsic LDA Accuracy

Applying LDA classification on our dataset required separating the set of throws from a given participant into discrete groups (classes). In the analysis presented in this paper we have focused on the problem of *Side* classification (Right vs. Left). In principle, the same analysis can be applied to the classification of *Elevation* (Up vs. Down) and or *Quadrant* (*Side* × *Elevation*). In these cases, however, because the effect of gravity on the ball’s elevation at impact depends on the flight duration, the ball velocity at release acts as a confound in the relation between throwing kinematics and ball elevation at impact.

As described in Section “Materials and Methods,” throws were grouped based on the coordinates of the position in which the ball landed on the targets-board. This choice allowed including most of the performed throws in the LDA training, even for poorly performing participants. Only the throws in which the ball landed in the gray separation region were indeed excluded from the LDA analysis. The number of excluded trials depended on the participant’s performance and ranged from a maximum of 24 trials to a minimum of 3, with an average (±SD) of 17 (±6) trials removed per participant. The total number of trials used to perform LDA analysis for each participant is given in **Table 1**.

The choice of grouping trials into discrete classes based on continuous variables can have implication for the intrinsic LDA accuracy. In fact, throws assigned to different classes may be closer to each other, if the corresponding ball’s arrival positions are close to the boundaries of the separation region, than throws assigned to the same class. To assess the effect of the dispersion of the ball’s arrival positions on classification performances, we performed LDA *Side*-classification using position and velocity of the ball at release as predictors. Once released, the ball moves according to the laws of motion along a defined parabolic trajectory. It follows that ball position and velocity at a single point in time, from release onward, univocally determine where the ball will land on the targets-board. The LDA classification based on these predictors should be then accurate at 100% level, so that deviations from perfect classification could provide a quantitative estimation of the effect of sub-optimally separated classes on the intrinsic LDA performances.

MEs of the ball landing side based on ball position and velocity at release range from 0 to 4.2 percent, less than 1 percent when averaged across participants. Individual MEs are listed in **Table 1**. The differences found between participants in the intrinsic accuracy of the LDA should be taken into account when comparing the predictability of the throwing kinematics across participants. To this end, we introduced an index that normalizes the ME based on body kinematics to the ME due to the dispersion of the ball’s arrival positions on the targets-board. In the following, when reporting results from the LDA applied to body-kinematics features we provide values for the *Misclassification Index* (MI), defined as:

$$MI_{BK}=(ME_{BodyKin}-ME_{Ball})/(1-ME_{Ball}),$$

where ME_BodyKin_ and ME_Ball_ indicate the MEs obtained running LDA respectively on body kinematics and ball position plus velocity at release. If the classes are well separated and the ME based on ball data is zero, MI_BK_ corresponds to the ME based on body kinematics. In contrast, if the ME based on body kinematics is equal to the ME based on the ball, indicating that the body kinematics is as informative as the ball kinematics at release, MI_BK_ = 0.

#### Spatiotemporal Predictability of Individual Throwers

The aim of the analysis presented in this section was to characterize whether the throwing kinematics allows disentangling the side in which the ball will land on the targets-board, both at a spatial (i.e., in terms of single body parts) and at a temporal level.

**FIGURE 6:**
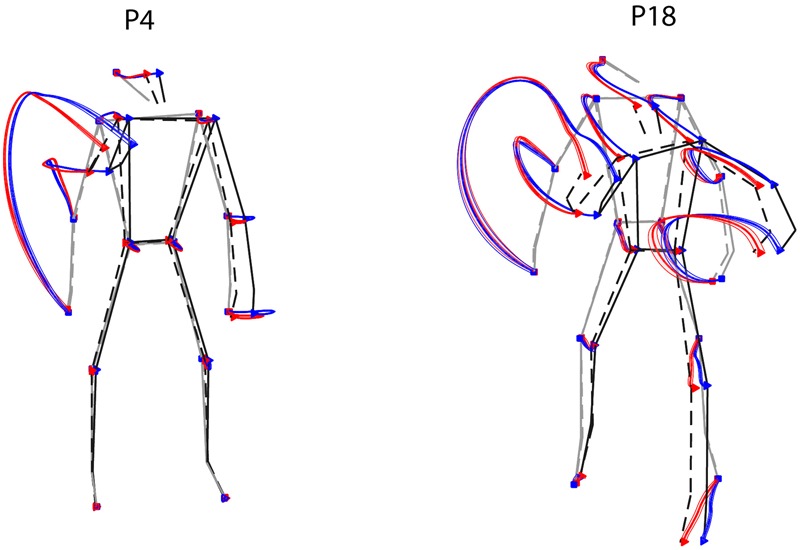
Examples of average kinematics by ball landing side. Average trajectories (thick solid lines), and corresponding standard errors (shaded areas), for throws to the right (red) and to the left (blue) are shown for two representative participants. The stick diagrams depict the average initial and final postures (gray and black respectively), for the right and left throws (dashed and solid respectively).

To a first approximation, one could address this issue by looking at the average differences in the joint-markers trajectories of throws to the left or to the right. **Figure 6** shows such a comparison for two representative participants, highlighting two main points. First, as expected (see Section Throwing Styles) the most dramatic differences between the kinematics leading to balls on different sides are associated with the *forward-projection* phase of the throwing arm. A second important point that emerges is related to the marked inter-individual differences. Trajectories corresponding to the ball landing on opposite sides tend in fact to diverge in the throwing arm at different stages for different participants (e.g., later for P18 than for P4). Furthermore, also depending on the participant, trajectories may diverge also for body parts other than the throwing arm (e.g., the left foot for P18).

Visualizing and inspecting differences in the individual average trajectories between left and right throws may give some hints about the kinematic cues that could provide useful predictions about the throwing outcome. However, the spatiotemporal stPCA + LDA approach described in Section “Analysis” was necessary to provide a quantitative characterization of the individual predictability of a thrower. LDA classification was run on the first N_PC_ spatiotemporal components that explain at least 98% of the total variance. The dimensionality of the predictors space, N_PC_, depends therefore on the specific participants and on the set of the kinematic variables included. The mean N_PC_ values (averaged across participants, joint markers and time steps) used for LDA for different combinations of joint-marker sets and time intervals are summarized in **Table 2**. Mean N_PC_ values range from 6 to 27, corresponding to original spaces with dimensionality between 60 (single joint-marker at one *time-decile*) to 1800 (e.g., one body-part with three joint-markers at the tenth *time-through* interval), demonstrating the efficacy of stPCA as a dimensionality reduction technique.

**Table 2:** Average number of stPCs components needed to account for 98% of the total variance and used for the LDA analysis.

	Time deciles (all) mean ± SD	Time through 1^st^ decile mean ± SD	Time through 10^th^ decile mean ± SD
Single joint-markers	6.0 ± 0.6	5.7 ± 0.6	18.8 ± 3.9
Body parts	9.1 ± 0.5	9.2 ± 1.4	27.2 ± 6.3


Averages are computed across participants, joint markers and time steps.

**FIGURE 7:**
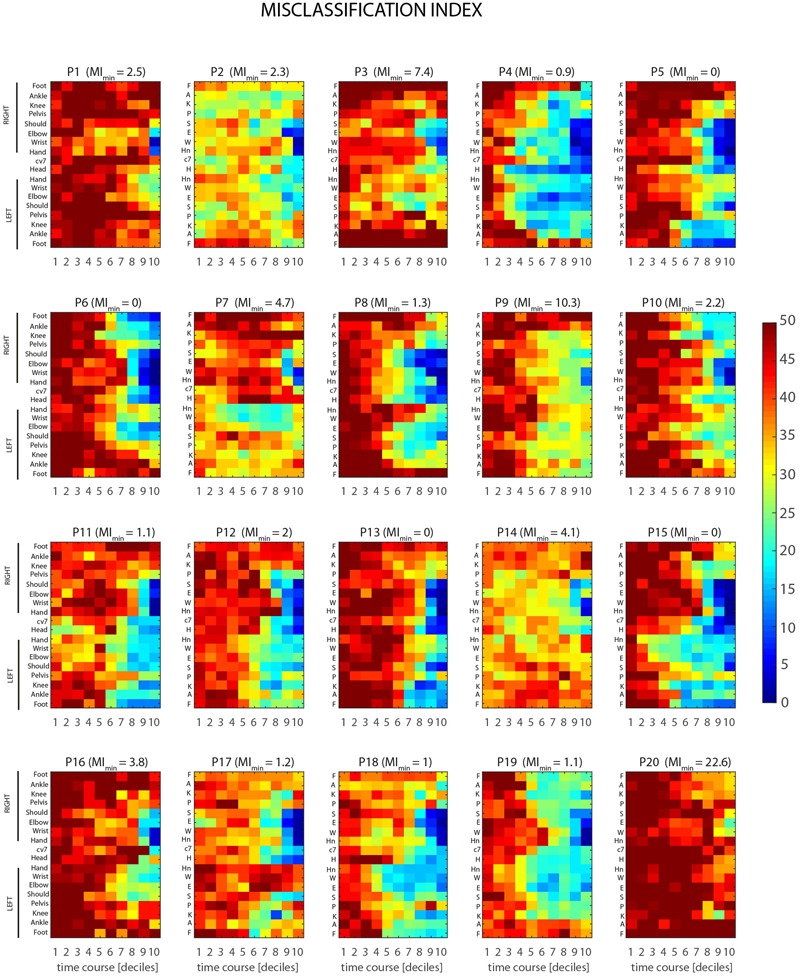
Individual spatio-temporal structures of predictability. Color maps represent the misclassification index, MI_BK_ (see Section Ball Flight Predictability based on Throwing Kinematics), as a function of joint-marker and time decile throughout the course of the throwing action, for all the twenty participants recruited in the study. Marked inter-individual differences show that different participants are characterized by different overall levels of predictability (MI_BK_ values on the top of each map gives the individual minimum value of MI_BK_ across joint-markers and time intervals). Furthermore, different participants become predictable at difference stages of the action, with relevant information provided by subject-dependent key joint-markers.

Results from stPCA + LDA analysis, for single joint-markers and the case of *time-decile* are shown in **Figure 7**. The color maps, one for each of the 20 participants recruited in the study, represent the MI, as defined in Section “Intrinsic LDA Accuracy” (MI_BK_), as a function of joint-marker and time decile throughout the throwing action course. The dataset reveals a considerable inter-individual variability in the predictability of the throwing action, both in its average level and in its spatiotemporal structure. Some participants are more predictable than others, reaching in fact different absolute minimum of the MI_BK_ across joints and times (values are given in **Figure 7**). Individual MI_BK_ minima range in fact from 0 to 22.6 (corrected percentage of misclassified trials), although on average participants display very low minima in MEs, i.e., 3.4 ± 5.2 (mean ± SD). Furthermore, the time intervals and joint-markers that provide information allowing for reliable predictions vary significantly across participants. This is clearly shown by the differences, found across individual maps, in the extent and shape of the bluish regions, which correspond to predictions accuracy higher than 80%.

**FIGURE 8:**
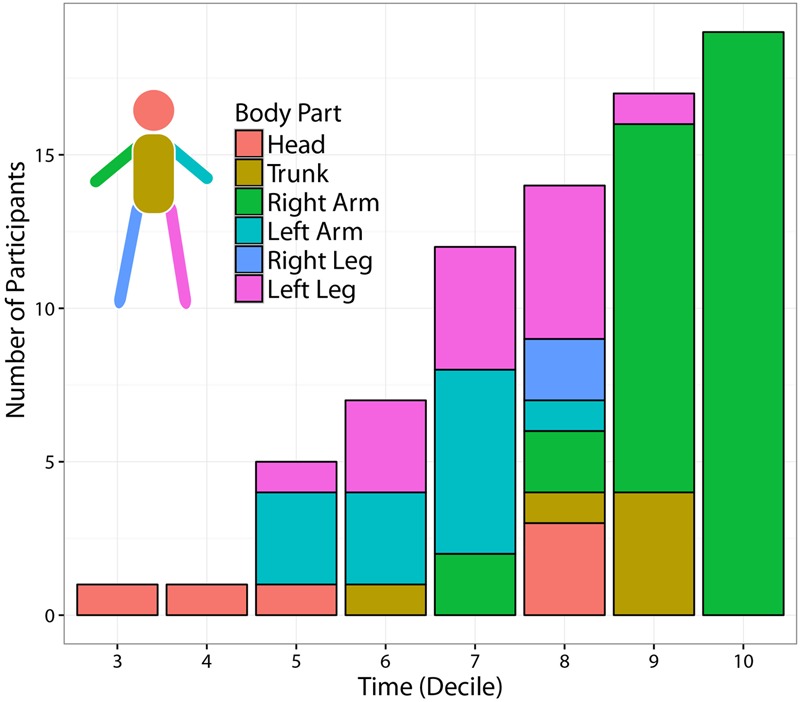
Temporal evolution of most informative body parts across participants. The figure displays the number of participants for which at least one joint-marker could predict the *Side* of the outgoing ball trajectory with at least 80% accuracy. The segmentation of each temporal bin shows the distribution, across participants, of the body segments including the most informative joint-marker. The number of predictable participants increases with time. Time modulates also the spatial origin of the most informative joint-marker. While the throwing arm provides the most informative cues towards the end of the action, other body parts –in particular the contralateral limbs– become more informative when moving back in time.

Despite the overall inter-individual differences, two important general results emerge. First, the throw’s outcome may become predictable with a reasonable accuracy (>80%) not only before ball release, but even before the *forward-projection* phase. Although for some participants informative cues about the outcome are not available until the very end of the action (e.g., P1, P7 P9, P14, P16, P20), most of the participants display informative kinematic cues already during the 6–7th deciles, i.e., 400–500 ms before ball release, and in few cases even earlier (e.g., P4, P18). Second, when early information is available, it is not necessarily provided by the throwing arm, but instead by different body segments that vary across participants. Interestingly, the body segments that deliver early information seems to be associated to specific throwing styles. For example, participants for which early predictions are based on the left lower leg joint-markers (e.g., P5, P11, P15) perform the throwing action with a prominent left step (see **Figures 3, 7**), and the same applies to participants delivering early information from the right lower leg (e.g., P6, P10, P19). An insightful summary of the inter-individual variability in the spatio-temporal structure of throwing predictability in given in **Figure 8**.

**Figure 8** displays the number of participants for which at least one joint-marker could predict the ball landing side, with at least 80% accuracy, as a function of time. In addition, for each temporal interval, the figure shows the distribution across participants of the body segments (i.e., subsets of contiguous joint markers) containing the most informative joint-marker. Not surprisingly, the number of predictable participants increases throughout the action course. Interestingly, the spatial origins of the most informative joint-marker is modulated by the stage of the action at which predictions are made. The throwing arm results to be the most reliable source of information only at the very end of action, i.e., during the 9–10th deciles. Moving back in time, useful information is instead provided by other body segments, in particular the limbs contralateral (left) to the throwing arm. This could possibly reflect the relevance of early kinematic features tuning the *forward-projection* phase.

#### The Role of Information Integration across Time and Body Parts

Results from Section “Spatiotemporal Predictability of Individual Throwers” provide insights on how early it is possible to predict the outcome of a throws and which are the body parts that, at different times throughout the action, provide the most reliable information. As a next step, we here look at the effect of integrating information both spatially (across different subsets of joint-markers) and temporally (across time intervals of different extents). We do this by comparing results from LDA applied on four different predictor spaces: *single joint-markers* and *body-parts* kinematics, both integrated either over *time-decile* or *time-through* temporal intervals (see Section Linear Discriminant Analysis for throwing outcome classification for details of the different datasets definition).

**FIGURE 9:**
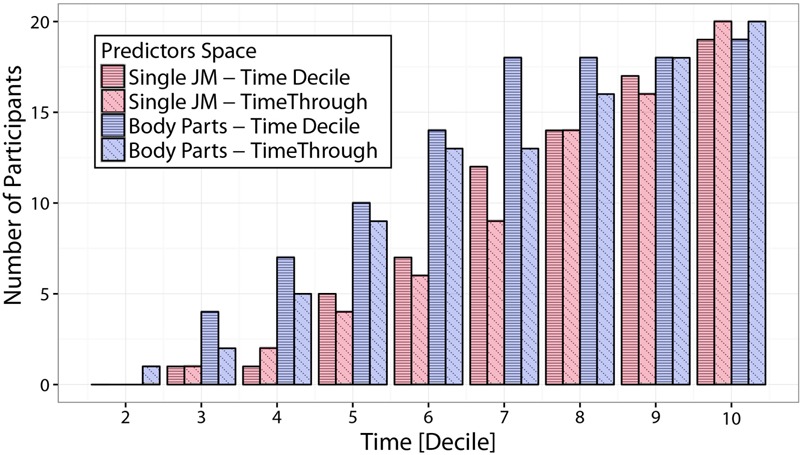
Comparison of predictability accuracies based on different predictors spaces. The figure displays predictions accuracy in terms of number of participants for which *Side* predictions can be made with an accuracy of at least 80% based on at least one of the spatial predictors (either a single joint-marker or a body part), at different temporal intervals. Predictions accuracies are compared for predictors spaces that differ in either the amount of spatial information integrated (single joint-markers vs. subset of joint-markers grouped in body parts), and/or of temporal information integrated (non-overlapping *time-deciles* vs. time intervals of increasing lengths, from movement onset to the actual time, i.e., *time-through*). Spatial integration of information from extended body districts significantly improves the accuracy of throws predictability, particularly at the early phases of the throwing action.

**Figure 9** shows LDA performances in terms of number of predictable participants, for the four different choices of spatial and temporal integration under consideration. Participants counts correspond to the number of participants for which *Side* predictions can be made with an accuracy higher than 80%, based on at least one of the spatial predictors (either a single joint-marker or a body part) for each temporal interval. Focusing on the comparison between *single joint-markers* and *body-parts* (red and blue bars respectively), it is evident that integrating information spatially to include multiple joints into body-parts results in a clear boost in predictability. This is particularly true for the early stages of the action. Looking for example at the *time-decile* results, it can be seen that integrating spatial information in such a fashion increases the number of “predictable” participants by more than 50% up to the 6th decile. On the other hand, looking at the effect of temporal integration it appears that integrating information across time (*time-through*, diagonal-dashed), does not result in higher prediction accuracies with respect to taking into account only the information within the specific time-decile (*time-decile*, horizontal-dashed), and in some cases may even entail loss in accuracy.

## Discussion

The focus of this study was to assess whether it is possible to predict the direction of a thrown ball based on advanced information from the whole-body kinematics of the thrower. In particular, we explored how early it is possible to make reliable estimates about the lateral direction (right versus left) in which the thrown ball will reach a vertical plane placed at 6 m from the thrower. In addition, we explored the spatial structure of the predictive information, namely what are the body parts from which it is possible to make the most accurate predictions, and how this spatial structure depends on time and on individual throwing strategies. The study represents an essential first step for establishing, in future studies, how human or robotic agents could take advantage of visual cues extracted from the observed biological motion in order to optimize interpersonal interaction strategies.

The study is based on the kinematics of throwing actions recorded from a sample of twenty non-expert participants that were asked to perform non-constrained overarm right-hand throws to hit one of four targets at 6 m distance. In order to have a representative sample of “typical” throwing patterns in the adult population, we posed no specific constraints on the previous sport exposure and experience when recruiting participants. We found a high level of heterogeneity in throwing styles, as each individual typically tended to adopt a specific throwing strategy over the course of the experiment (**Figure 3**). This is not completely surprising, given that besides factors such age, gender and intrinsic inter-individual differences, the previous exposure to different sports and different skill levels are likely to play a crucial role in determining individual preferences for a specific throwing strategy. Similar considerations hold in the context of other ecological scenario, such as unconstrained ball catching (Cesqui et al., 2012).

To quantify the spatiotemporal structure of a throwing action predictability, we developed a novel method based on a combination of dimensionality reduction and machine learning techniques, which was applied to different sets of kinematic variables. This approach led to several significant results. First, we found a considerable inter-individual variability in the predictability of the throwing action, as some participants were more predictable than others in terms of launch direction. Furthermore, different participants became predictable at different times across the throwing action duration. For most participants accurate (>80%) predictions can be made as early as 400–500 ms before ball release, and for a few participants even earlier (see **Figure 8**). This implies that a hypothetical human or robot catcher could in principle improve interceptive performance of genuine throws, e.g., by reaching in advance the most likely region of ball arrival, before being able to acquire more refined information from the ball flight. Nonetheless, the extent to which this holds critically depends on the agent’s ability to read out the information available, something that needs to be tested with dedicated studies. Moreover, this conclusion would not extend to the case of deceptive throws, in which the kinematics of the throwing action is intentionally and skillfully decoupled from the ball’s trajectory (Güldenpenning et al., 2017).

Second, our spatial analysis showed that the throwing arm delivers, as expected, accurate information about the outgoing ball trajectory, but only in the very last phase of the throwing action, during the *forward-projection* phase, i.e., at 100–200 ms before ball release. When moving back in time, the body parts providing informative cues shift from the throwing arm to other body segments, typically the trunk and the left limbs (contralateral to the throwing arm). With respect to this latter result, it is interesting to mention that previous studies from the sport science literature have described the role of contralateral arm for increasing the accuracy of a throw (Bartlett et al., 1996; M.C.C., 1976) or a kick (Bezodis et al., 2007). It seems therefore reasonable to speculate that, when the contralateral limbs are employed for stabilizing the throwing arm forward-projection, they could retain information about this phase in advance.

Third, we found a considerable inter-individual variability in the spatio-temporal structure of the information relevant for making reliable predictions. In fact, the spatial origin of early advanced information varies from participant to participant (see **Figure 8**). Thus, for a catcher to be able to take advantage of early information from the body kinematics, it would be probably necessary to have some *a priori* knowledge of the most informative body parts that characterize the specific thrower, so as to direct the attention toward these body parts early on.

Interestingly, a qualitative comparison between the spatiotemporal structure of predictability (**Figure 7**) and the individual throwing styles (**Figure 3**) suggests a possible link between the two. Participants who perform the throwing action with a prominent left (right) step tend to deliver early information mainly via the kinematics of the lower left (right) leg. A quantitative assessment of this potential correlation between throwing styles and spatiotemporal structure of predictability would require a formal, data-driven definition, of individual throwing styles. Although this is beyond the scope of the present study, it is important to notice that the observed link may have crucial implications. In fact, it suggests that, in a possible interactive scenario, a catcher that has knowledge of the throwing style of the opponent, would know in advance where to look for extracting more efficiently advanced information, and therefore for maximizing interception performances.

Finally, looking at how prediction accuracies are modulated by spatial integration of advanced information from the throwing motion (by performing LDA classification on different predictors spaces), we found that integrating information across multiple joint-markers is of clear advantage for boosting prediction accuracies. This result is in line with previous findings highlighting how anticipation skills are associated with a “global” perceptual processing of kinematic information rather than with localized information (Huys et al., 2009; Smeeton and Huys, 2011).

Integrating information through the whole course of the action, instead, does not seem of clear advantage for increasing predictability accuracies. In some cases, temporal integration may even result in reduced predictions accuracies. This pattern of results may be explained by two different effects. A possibility is that taking into account information over extended time intervals may blur key relevant information, because of cumulating non-informative variance across time. A second possibility is that the observed loss in prediction accuracy is a result of the higher dimensionality of the predictors space, which introduces limitations in the intrinsic LDA accuracy (Fukunaga, 1990).

The present findings will guide future experimental studies in which we plan to explore sensorimotor strategies involved in interactive throwing and catching tasks. Indeed, the main aim of the current study was to provide a quantitative characterization of the information embedded in the throwing kinematics about the outgoing ball trajectory. The gathered knowledge about the spatiotemporal structure of the information available to a potential observer is in fact functional to the design of tailored experimental protocols that will assess whether and how human observers are able to read out the relevant information and, if this is case, whether and how the extracted information is used in interaction performances. While this principled approach has been previously introduced for the study of predictions from the kinematics of reach-to-grasp movements in the context of social neuroscience (Ansuini et al., 2014, 2015a), it is entirely novel with respect to previous works on the role of advanced information in more complex whole-body movements like throwing actions.

Previous studies provided robust evidence for the human ability to read out advanced information from throwing, hitting and kicking kinematics. In particular, these anticipatory skills have been shown to be higher for elite-sport players than for non-experts (Abernethy, 1990a; Aglioti et al., 2008; Diaz et al., 2012) and to be associated with a refined task-specific knowledge that allows experts attending the most informative task-specific kinematic cues (Abernethy and Russell, 1987; Ward et al., 2002). Although the specific nature of the most informative kinematic cues is dependent on task constraints (Müller et al., 2006), some previous results are in line with our findings. In particular, it has been shown that expert players are not only able to use information from the throwing, hitting or kicking end-effector (e.g., hand, racket or foot) at times close to impact, but also earlier kinematic cues from more proximal or different distal body parts (Abernethy et al., 2001, 2008; Nagano et al., 2004). Interestingly, several studies have highlighted how the picking-up of advanced information is associated with a preparatory motor behavior, like stepping in the predicted direction (Ranganathan and Carlton, 2007; Renshaw et al., 2007), and/or with earlier onset of the interceptive movement (Panchuk et al., 2013; Stone et al., 2017). In continuity with these results, we plan, as a next step, to assess the extent to which similar observations hold for catchers facing non-experts performing unconstrained overarm throws, and whether adaptation and attunement to individual throwing styles is present.

Beside the specificity of the throwing/catching scenario, the current study fits in the wider context of social neuroscience and human-robot interaction. It is indeed well established that humans, when observing an action, are extremely good at reading out subtle kinematic cues not only to infer actor’s attributes –such as emotional states (Pollick et al., 2001), gender (Barclay et al., 1978) or identity (Richardson and Johnston, 2005)–, but also to make specific predictions about the actor intention (Sartori et al., 2011; Cavallo et al., 2016) or the action outcome (Ansuini et al., 2015b). Early predictions formulated during action observation are indeed revealed in gaze behavior strategies functional to test the foreseen stages of the observed movement (Flanagan and Johansson, 2003; Donnarumma et al., 2017). A series of recent studies have furthermore highlighted how informative kinematic cues are spontaneously modulated to facilitate the discriminability of the performed action, so to render interpersonal communication more efficient (Vesper and Richardson, 2014; Candidi et al., 2015). Our work represents a complementary contribution in this field by providing a tool for establishing, in an unbiased fashion, the spatiotemporal structure of the relevant information embedded in a specific action about its own outcome. This knowledge could be then used in future studies to explore which aspects of the information available are effectively accessed and used in non-verbal interpersonal communication, for example by implementing occlusion paradigms and/or by motoring interceptive actions and eye movements during interactive throwing and catching tasks.

Importantly, the methodology that we have introduced for deciphering the spatiotemporal structure of the information embedded in the throwing kinematics about the outgoing ball trajectory, can be applied to other categories of whole-body actions involved in the interaction between two or multiples agents. As such, it provides a valuable tool to explore interpersonal communication strategies based on body kinematics in a large variety of interactive tasks. Furthermore, the insights gained from observed human behavior can provide a significant contribution in human-robot interaction research, both by guiding the design of better predictable robots, and by addressing potential strategies for artificially reading out relevant advanced information from human kinematics.

Despite the novelty of the results and the potential future applications of the methodology, our study is subject to some limitations. From the methodological point of view, the combination of dimensionality reduction and classification techniques that we have used may become less reliable if the dimensionality of the predictor space grows too high with respect to the dimensionality of the dataset used to train the classifier. If, as we did, we fix a given degree of accuracy in the stPCA reconstruction (fixing a VAF threshold), the dimensionality of the predictor space depends on the kinematics taken into considerations. For example, when considering multiple joint-markers across the whole duration of the throwing action, the number of stPCs required to have a VAF above 98% could grow above 30. On the other hand, fixing the dimensionality of the stPCs space would correspond to different degrees of reconstruction accuracy for different subsets of kinematic variables considered. While the high dimensionality of the stPC representation may reflect an intrinsic and thus unavoidable feature of the data, it is also possible that there exists a more compact representation of the spatiotemporal variability of whole body kinematics. Rather than being only superimposed over a fixed time-interval, individual stPCs may also be shifted in time (d’Avella et al., 2003). The use of time shifting might allow to better capture timing modulation in the throwing action and could achieve a better reconstruction accuracy in a stPCs space of lower dimension (Chiovetto et al., 2016).

Another limitation of the study is linked to the choice of recruiting non-expert participants. While this choice was meant to provide a broad picture of the readability/predictability of individuals without a specific expertise in a complex whole-body action like an overarm throw, it is likely that such a population is characterized by a lower “signal-to-noise” (i.e., by larger amount of non-informative variance in the movement kinematics) with respect to expert throwers. An additional drawback of having non-expert throwers as participants is related to their average poor performances (ball landing positions were in most cases not clustered around the aimed targets) and the associated need to group throws into discrete groups (the side) on the base of continuous variables (the ball landing position coordinates). It would be interesting in future studies to apply the same methods as to characterize the spatiotemporal structure of the predictability of expert throwers with respect to the outgoing ball trajectory.

## Conclusion

In this study we have provided a quantitative estimate of the accuracy that it possible to achieve when predicting whether a thrown ball will flight right or left, as a function of different visual cues from the whole-body throwing kinematics. We achieved this by introducing a novel method based on a combination of dimensionality reduction and machine learning techniques. The results provide novel insights into the spatiotemporal structures and inter-individual variability of the visual cues potentially available for predicting the outgoing ball trajectory. The results further provide preliminary evidence for a relationship between the spatiotemporal structure of the information available and the individual throwing styles.

## Author Contributions

All authors conceived the study. AM, AD, and Ad’A designed the experiment. AM, AD, BC, and MR implemented the experimental setup. AM and AD conducted the experiments. AM analyzed the data. All authors discussed and interpreted the results. AM drafted the manuscript and all authors provided inputs to its final version.

## Conflict of Interest Statement

The authors declare that the research was conducted in the absence of any commercial or financial relationships that could be construed as a potential conflict of interest.

## Footnotes

1 http://optitrack.com/products/natnet-sdk/
