# Supplementary material for: Where Are You Throwing the Ball? I Better Watch Your Body, Not Just Your Arm!
Source: Front Hum Neurosci. 2017 Oct 30;11:505. doi: 10.3389/fnhum.2017.00505 (PMC5674933; doi:10.3389/fnhum.2017.00505)
Supplement: Supplementary file 1 [file Data_Sheet_1.docx]

Supplementary Material

**Where are you throwing the ball?**

**I better watch your body, not just your arm!**

Antonella Maselli^*^, Aishwar Dhawan, Benedetta Cesqui, Marta Russo, Francesco Lacquaniti, Andrea d’Avella

*** Correspondence:** Corresponding Author: [a.maselli@hsantalucia.it](mailto:amaselli@hsantalucia.it)


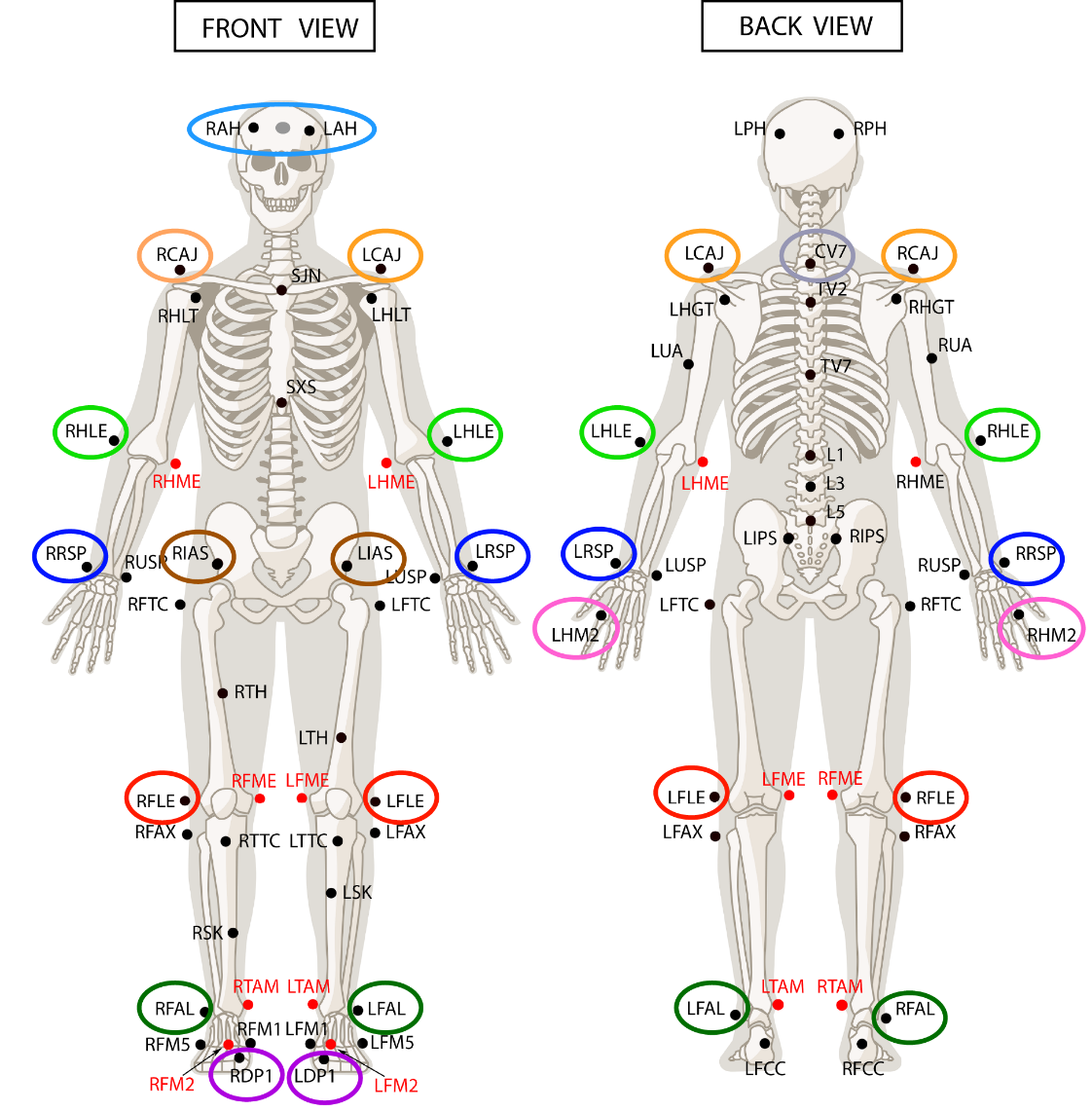


Supplementary Figure 1. Marker set used for motion capture. Eight calibration markers (red) were removed for the actual recordings. The markers used for the analysis presented in this studies are indicated with circles (colors corresponding to those used in Figure 3 in the main text manuscript).


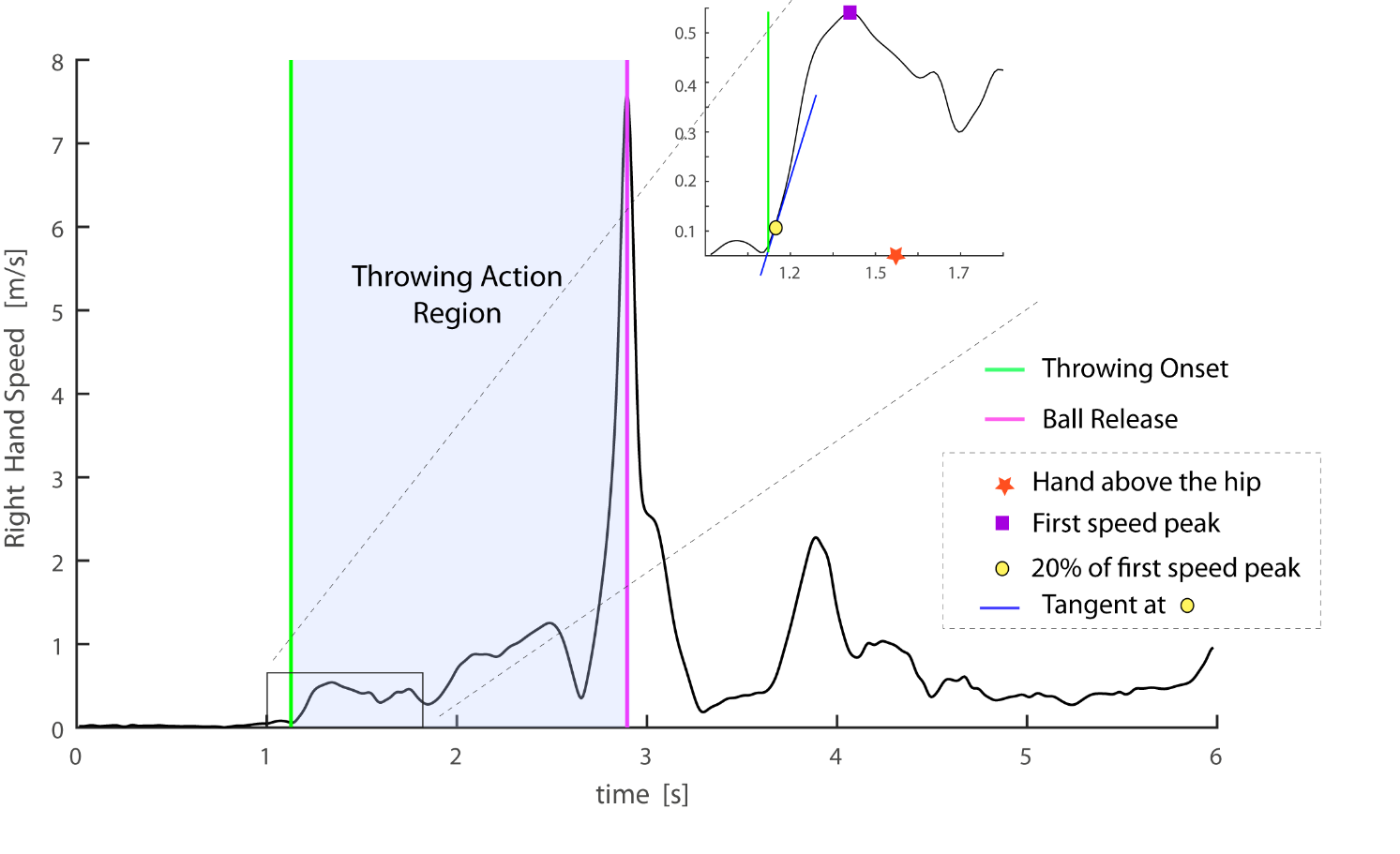


Supplementary Figure 2. Identification of action onset and ball release in a representative trial. Both the throwing action onset (green line) and the ball release time (magenta line) are detected from key features in the speed profile of the throwing hand (black line). The red star symbol represents the frame at which the right hand rises 5 cm above the right hip. The shaded region delimits the temporal duration of the throwing action within the trial.


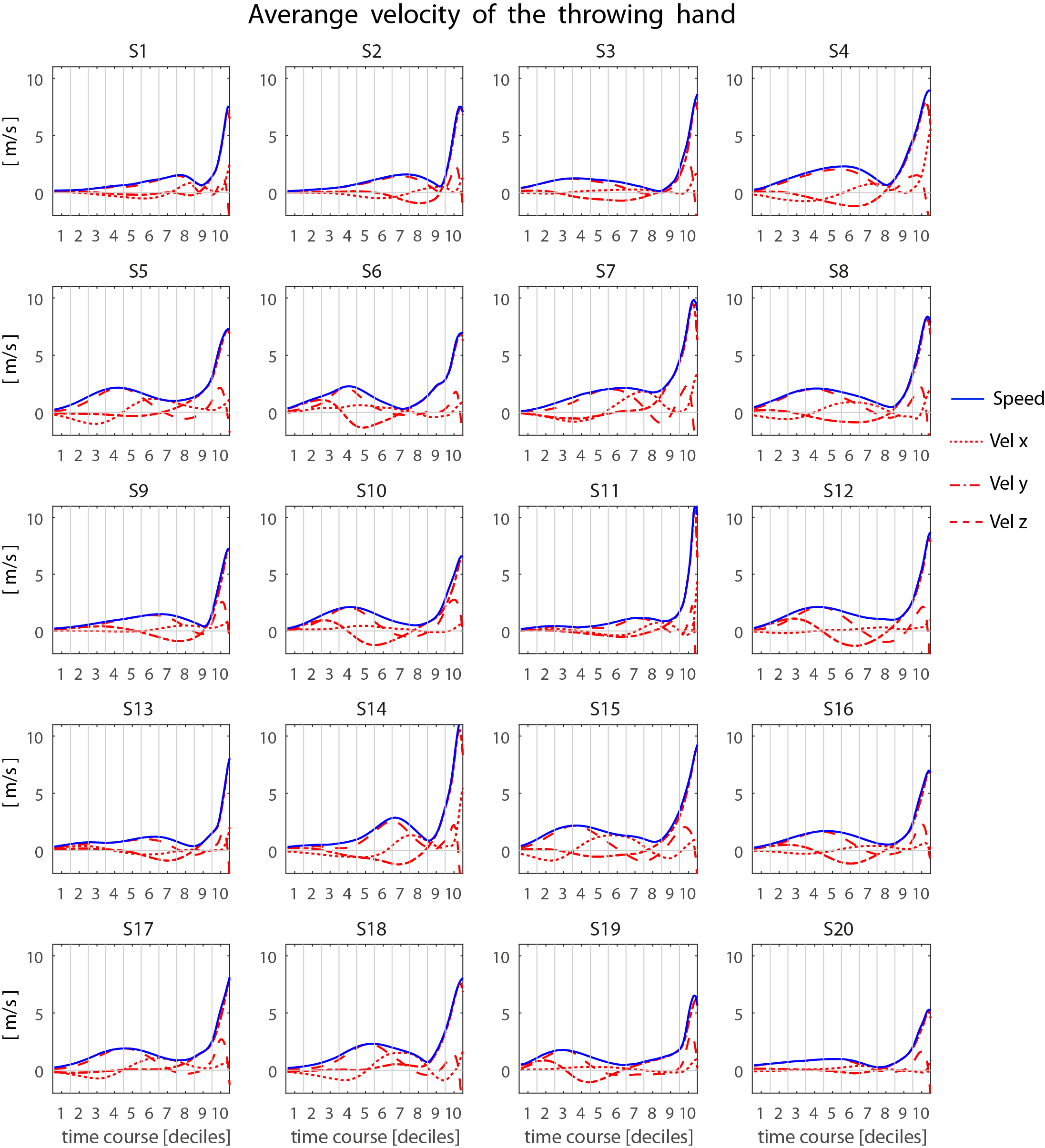


Supplementary Figure 3. Individual average velocity and speed profiles of throwing hand. For each of the 20 participants recruited in the study, the figure show velocity (red dotted, dotted-dashed and dashed lines respectively for the x, y and z components) and speed profiles (solid blue lines) averaged across all trials, irrespectively of the intended target.

**Table S1. Dimensionality of the kinematics included for different choices of time intervals and joint-markers combinations considered in the analysis.**

| **Joint-markers Set** | **Time-Decile (1÷10)** | **Time-Through (1÷10)** |
| --- | --- | --- |
| Single Joint-Marker | 60 | 60÷600 |
| Head | 120 | 120÷1200 |
| Trunk | 240 | 240÷2400 |
| Right and Left Arm, Right and Left Leg | 180 | 180÷1800 |
